# Dual Tuning of Biomass-Derived Hierarchical Carbon Nanostructures for Supercapacitors: the Role of Balanced Meso/Microporosity and Graphene

**DOI:** 10.1038/srep15936

**Published:** 2015-10-30

**Authors:** Zhengju Zhu, Hao Jiang, Shaojun Guo, Qilin Cheng, Yanjie Hu, Chunzhong Li

**Affiliations:** 1 Key Laboratory for Ultrafine Materials of Ministry of Education, School of Materials Science and Engineering, East China University of Science & Technology, Shanghai 200237, China; 2 Department of Materials Science & Engineering, College of Engineering, Peking University, Beijing 100871, China

## Abstract

Rational design of advanced carbon nanomaterials with a balanced mesoporosity to microporosity is highly desirable for achieving high energy/power density for supercapacitors because the mesopore can allow better transport pathways for the solvated ions of larger than 1 nm. Inspired by the inherent meso/macroporous architecture and huge absorption ability to aqueous solution of auricularia biomass, we demonstrate a new biomass-derived synthesis process for the three-dimensional (3D) few-layered graphene nanosheets incorporated hierarchical porous carbon (GHPC) nanohybrids. The as-prepared GHPC nanohybrids possess a balanced mesoporosity to microporosity with much improved conductivity, which is highly desirable for achieving high energy/power density for supercapacitors. As we predicted, they delivered a high specific capacitance of 256 F g^−1^ at 1 A g^−1^ with excellent rate capability (120 F g^−1^ at 50 A g^−1^) and long cycle life (92% capacity retention after 10000 cycles) for symmetric supercapacitors in 1 M H_2_SO_4_. Based on the as-obtained carbon materials, a flexible and all-solid-state supercapacitor was also assembled, which can be fully recharged within 10 s and able to light an LED even under bended state. Such excellent performance is at least comparable to the best reports in the literature for two-electrode configuration under aqueous systems.

Climate change and the decreasing availability of fossil fuels require the researcher to develop better energy conversion and storage technique towards future sustainable and renewable resources^1^,^2^,^3^. Supercapacitor is a new class of energy storage device with high power energy, good reversibility and long cycle life^4^,^5^,^6^. Traditionally, activated carbons and carbon nanomaterials with many micropores are widely used as electrode materials for supercapacitor applications because of their high specific surface area (2000–3600 m^2^ g^−1^)^7^,^8^. However, their relatively low power performance for supercapacitor is still a big issue, far away from satisfactory from the viewpoint of perusing much higher energy/power density and much faster storage device in modern society, due to their intrinsically high fraction of microporosity and tortuous pore structure^9^. This close to 100% micropore in activated carbon and well-established carbon nanomaterials can partially restrict the pore accessibility of the electrolyte ions especially at high current densities because the size of the solvated ions (usually larger than 1 nm) is larger or comparable with the size of micropores^10^,^11^. In this regard, despite the great potential of these carbon matrials in providing large surface area for supercapacitor, they can only achieve a specific capacitance in the range of 100–300 F g^−1^ in aqueous electrolytes with a relatively poor rate capability^7^. Considering this, the moderate introduction of mesopores on advanced carbon nanomaterials may open an advanced approach to achieve better capacity and rate ability for supercapacitor application by shortening the ion transport pathways or accelerating ion transport rate^12^. Therefore, a concept on rational design of specially designed carbon nanomaterials with a well balance between mesoporosity and microporosity is highly desirable to simultaneously achieve high energy/power density, yet a great challenge to date^13^. Furthermore, another important factor for enhancing supercapacitor performance that we cannot neglect is that the carbon nanomaterials should have very high conductivity for accelerating electron transfer^14^,^15^. Considering that after even creating a good balanced micropore to mesopore ratio on carbon nanomaterials, their conductivity will partly sacrifice, the additional incorporation of graphene with ultrahigh conductivity into porous carbon nanomaterials framework forming a three-dimensional (3D) conductive network may provide another interesting, yet necessary route for further enhancing the supercapacitor performance^16^,^17^. However, to the best of our knowledge, it is still a very challenge to develop novel carbonaceous materials with a good balance between the conductivity and proper micro to mesopore ratio for high performance supercapacitors.

Auricularia, a species of edible mushroom, is one of the most abundant fungi in nature, which are cultivated more than 460000 ton per year all over the world. One natural phenomenon is that auricularia can absorb amounts of various solutions up to 160 times its own weight with a huge volume expansion after soaking in aqueous solutions (Figure S1a). Inspired by their inherent meso/macroporous architecture and huge absorption ability to solution, herein, we demonstrate a new concept that 3D graphene incorporated hierarchical porous carbon (GHPC) nanohybrids have been successfully synthesized by immersing auricularia into exfoliated graphite oxide (GO) aqueous solution, then hydrothermally carbonizing the as-prepared auricularia/GO composite and finally activating the porous carbon/GO hybrid using KOH. The optimized GHPC nanohybrids exhibit high specific surface area (up to 1723 m^2^ g^−1^), rich mesoporosity (~75%) and large pore volume (up to 1.85 cm^3^ g^−1^). Such particular structure can not only provide better transport pathways for solvated ion due to the introduction of more mesopores, but also accelerate electron transport rate because of incorporated graphene into 3D porous carbon (Fig. 1), finally leading to a high specific capacitance of 256 F g^−1^ at 1 A g^−1^ and 120 F g^−1^ at 50 A g^−1^ with a long cycle life (92% retention after 10000 cycles) in 1 M H_2_SO_4_ for symmetric supercapacitors. Such excellent performance is at least comparable to the best results reported to date for two-electrode configuration in aqueous solution.

## Results

The typical preparation process for GHPC nanohybrids is illustrated in Figure S1b. The microstructure of the dried auricularia was first characterized, which fully absorbed distilled water and then through a freeze-drying process. We found that the inherent rich porous network in the cross section of auricularia has been well-preserved (Figure S2). It should be noted that on the outer surface of auricularia, an undulated compact structure with many wrinkles and tentacles can also be observed (Figure S3a,b), which endows the auricularia with huge volume swelling properties^18^. Based on these features, in the second step, we immersed −the dried auricularia into exfoliated GO aqueous solution. It can be seen that after the immersion, the exfoliated GO nanosheets have been well-anchored on the tentacles, as shown in Figure S3c,d. After that, through a simple hydrothermal carbonization and the subsequent activation process of the auricularia/GO composite, the GHPC nanohybrids can be obtained. Figure 2a shows the scanning electron microscopy (SEM) image of GHPC nanohybrids. An interconnected network of submicrometer-sized macropores can be observed. The diameter of the macropores is around 300 nm, being in good agreement with the observation of transmission electron microscopy (TEM) in Fig. 2b. The white circle labeled in Fig. 2b was further magnified to investigate the pore wall structure, showing an obvious mesoporous texture (Fig. 2c). In addition, several dark black stripes (white arrows) can be assigned to graphene, confirmed by high-resolution TEM images of Fig. 2d,e. The interplanar spacing of ~0.33 nm shown in Fig. 2d,e should correspond to the (002) plane of the graphitized carbon (*i.e.* graphene), which consists of few-layer graphene nanosheets. As a comparison, the hierarchical porous carbon (HPC) was also synthesized using the same strategy without the addition of GO (Fig. 2f,g). No obvious lattice fringe is observed in Fig. 2g (denoting the white circle in Fig. 2f). These results indicate our strategy is successful to realize the introduction of graphene into biomass-derived HPC framework, which will greatly improve their electrical conductivity.

Figure 3a shows the nitrogen adsorption/desorption isotherms of the GHPC, HPC and KOH-activated porous graphene (labeled as PG). GHPC shows a combined I/IV type adsorption-desorption isothermal with a steep increase in N_2_ adsorption at low pressure due to micropore filling. Meanwhile, a wide and pronounced hysteresis loop in GHPC reveals well developed mesopores, indicating that GHPC possesses a hierarchical porous structure. The GHPC exhibits a Brunauer-Emmett-Teller (BET) specific surface area of 1723 m^2^ g^−1^, which is much higher than PG (970 m^2^ g^−1^), but lower than HPC (2120 m^2^ g^−1^) due to its high mesopore-to-micropore ratio. Figure 3b depicts the pore size distribution (PSD) determined by desorption data using the Barrett-Johner-Halendar (BJH) model. The average pore size is 4.3, 2.8 and 2.7 for GHPC, HPC and PG, respectively. Notably, a richer mesoporosity has been created (~75%) for GHPC with a large pore volume (up to 1.85 cm^3^ g^−1^ *vs.* 1.48 cm^3^ g^−1^ of HPC). Such intriguing pore structure and high surface area are very important for achieving high energy density and power density for supercapacitors.

Figure 3c gives the X-ray diffraction (XRD) patterns for the three samples. The PG exhibits a strong peak at 24.5° and a weak peak at 43.3°, suggesting its partial re-stacking and aggregation. As for GHPC, it only shows a weak diffraction peak at 24.5° like HPC, which further indicates few-layer graphene nanosheets are well-dispersed into HPC^19^. In addition, a large intensity increase in the low-angle scatter of GHPC is confirmed by Figure S4, which further suggests the presence of micropores and mesopores. The carbon quality of the three samples is evaluated by Raman spectroscopy. As shown in Fig. 3d and Figure S5, all samples exhibit a broad disorder-induced D-band (~1345 cm^−1^) and in-plane vibrational G-band (~1590 cm^−1^). The ratio of the integral intensity (I_D_/I_G_) can be estimated to 1.24, 1.47 and 1.14 for GHPC, HPC and PG, respectively. It can be concluded that the GHPC possesses higher graphitization degree than HPC, indicating a better electrical conductivity due to the incorporation of graphene. Furthermore, we found that the GHPC contained rich heteroatoms of N and O elements mainly from self-doping of auricularia and KOH-activation process, respectively. The content of N and O can be estimated to ~2.5 wt% and 12.1 wt%, respectively, determined by X-ray photoelectron spectroscopy (XPS), as shown in Figure S6. The ratio of N/C in GPHC has been further estimated to 2.2% by elemental analysis (Elementar Vario EL III, Germany), which is closed to XPS result (2.9%). These heteroatoms will induce additional pseudo-capacitance as well as enhanced wettability between electrode materials and electrolyte^20^,^21^,^22^.

The electrochemical performance of the GHPC with different graphene content, HPC and PG was evaluated by assembling these materials into symmetric two-electrode system in 1 M H_2_SO_4_ aqueous solution. Figure S7 shows the specific capacitance of GHPC with different graphene content for supercapacitors using three-electrode test system. We found that the optimized weight ratio of auricularia to GO for GHPC-based supercapacitor is 30:1 and the capacity contribution from graphite paper as substrate is negligible (Figure S8). The cycle voltammograms (CVs) of the optimized GHPC nanohybrids within the potential range of 0–1.0 V at the scan rate of 20–400 mV s^−1^ are shown in Fig. 4a. All the curves display a quasi-rectangular and symmetric shape, even at a very high rate of 400 mV s^−1^, implying a quick dynamics with high-power behaviors. Furthermore, a pair of small peaks can also be observed in CV curves under a three-electrode test system (Figure S9a), attributed to the presence of numerous heteroatoms, i.e. N and O, confirmed by XPS evaluation (Figure S6)^23^. Figure 4b presents the galvanostatic charge/discharge curves of the optimized GHPC at current densities of 1–50 A g^−1^. Each charging curve is well-symmetrical to their corresponding discharging counterparts. Figure 4c shows the specific capacitance as a function of current density for the optimized GHPC, HPC and PG. All the specific capacitances are calculated from the discharge curve (see Experimental Section for more details). At a low current density of 1 A g^−1^, the specific capacitance as high as 256 F g^−1^ can be achieved for the optimized GHPC, which is much higher than HPC (200 F g^−1^) and PG (75 F g^−1^). Even at a high current density of 50 A g^−1^, the capacitance of GHPC still retains 120 F g^−1^, while only 75 F g^−1^ and 26 F g^−1^ are maintained for HPC and PG, respectively. The similar results could also been observed in three-electrode system (Figure S9). Such fascinating electrochemical performance is superior or comparable at least to the best results for carbon-based nanomaterials under two-electrode test system in the literature (detailed comparison in Table S1), such as that of the silk-derived microporous carbon nanoplates (120 F g^−1^ at 52.5 A g^−1^)^24^, PPy-derived hierarchical porous carbon (182.3 F g^−1^ at 30.0 A g^−1^)^9^, and bacterial cellulose-derived carbon nanofiber networks (125 F g^−1^ at 50.0 A g^−1^)^25^. Our GHPC also exhibits long cycle life for supercapacitors with ~92% retention of its initial capacity after 10000 cycles at a charge-discharge current density of 10 A g^−1^, as shown in Fig. 4d. The Figure S10 shows Ragone plot of GHPC in two-electrode system, which exceeds a lot of reported carbon materials (Table S2). Such impressive results indicate our as-obtained GHPC nanohybrids are a very promising supercapacitor electrode material, especially for high-rate applications.

## Discussion

**Figure 1:**
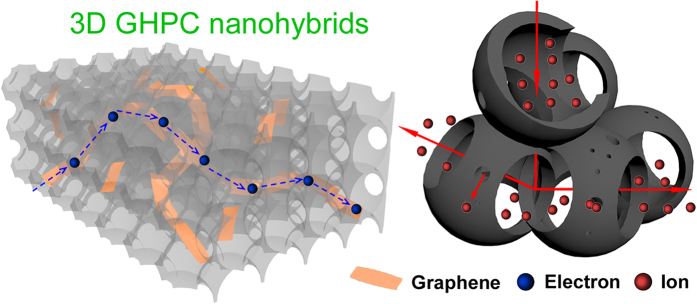
Schematic illustration of our new concept on GHPC nanohybrids for enhancing supercapacitor performance.

**Figure 2:**
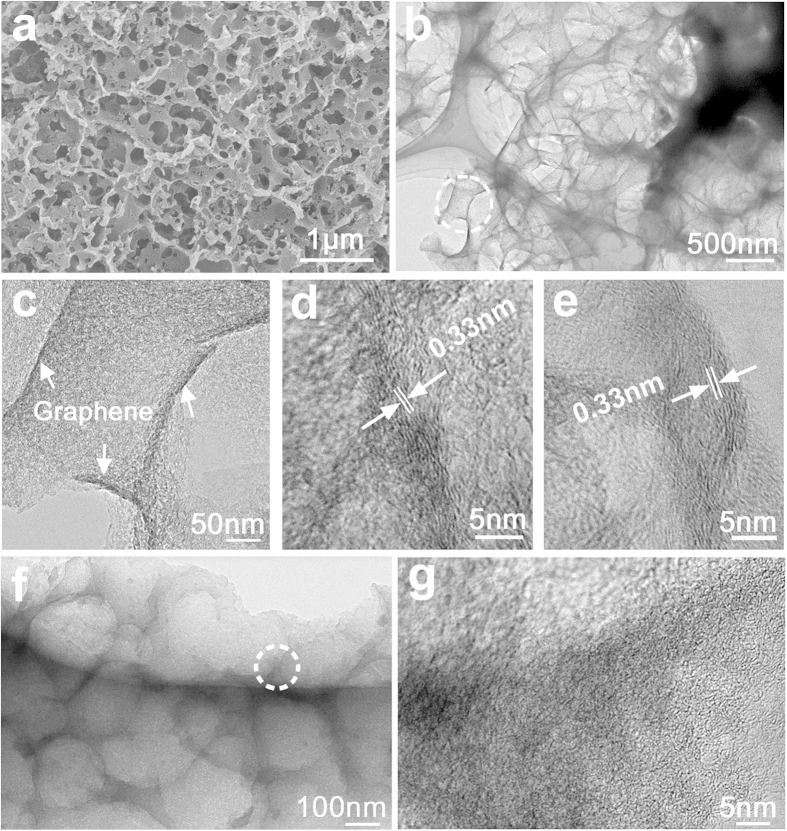
(**a**) SEM image, (**b**) low-, (**c**) high-magnification, and (**d**,**e**) high-resolution TEM images of the GHPC, (**f**,**g**) TEM images of the HPC.

**Figure 3:**
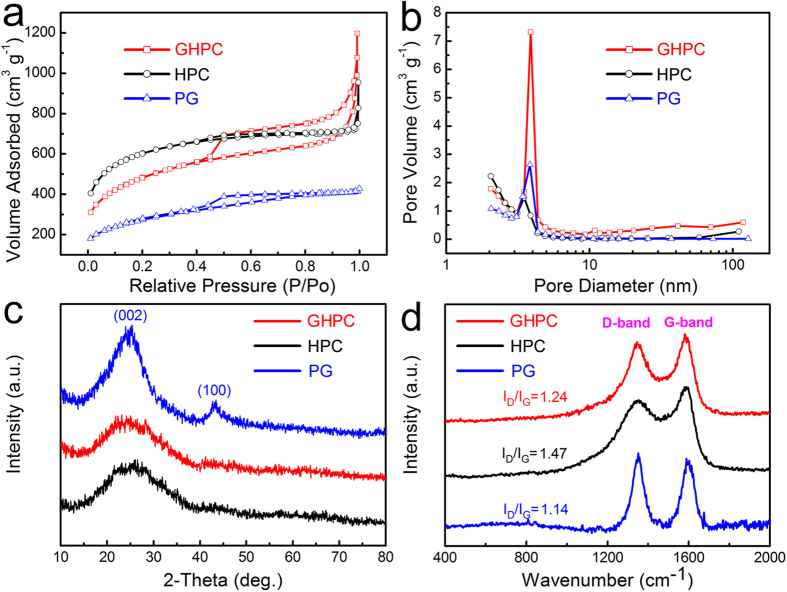
(**a**) Nitrogen adsorption/desorption isotherms and (**b**) the corresponding pore-size distribution curves using BJH model, (**c**) XRD patterns, and (**d**) Raman spectra of GHPC, HPC and PG.

**Figure 4:**
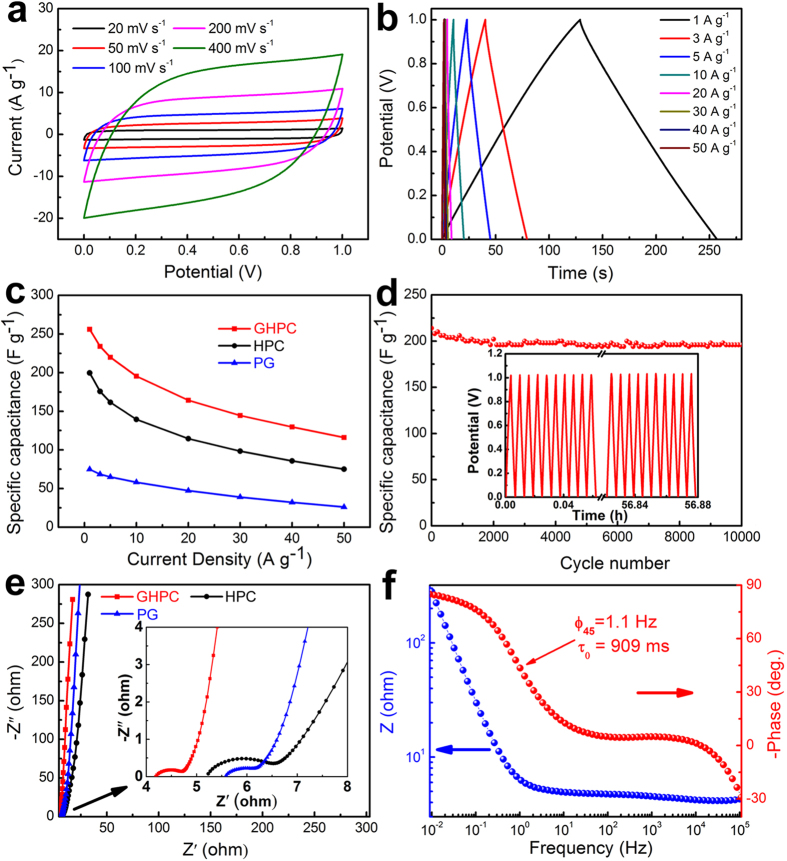
Electrochemical performances evaluated by symmetrical two-electrode supercapacitors in 1 M H_2_SO_4_ aqueous solution. (**a**) CV curves of the GHPC at various scan rates, (**b**) galvanostatic charge/discharge curves of the GHPC under different current densities, (**c**) the specific capacitance for GHPC, HPC and PG as a function of current density, (**d**) cycling stability of the GHPC, (**e**) the Nyquist plots of the GHPC, HPC and PG, (the inset showing expanded high-frequency regions), (**f**) the Bode plot of the GHPC.

We think that several important characteristics of our newly design of GHPC may contribute their excellent electrochemical performance for supercapacitor application. (a) The 3D hierarchical porous structure in GHPC can not only ensure the rapid ion diffusion by reducing the diffusion pathways, in which macroporous frameworks serve as ion-buffering reservoirs, mesoporous walls ensure smaller ion-transport resistance and microporous textures accommodate charges, but also provides 3D conductive networks. Notably, compared with the previous work in porous carbon nanomaterials, more mesopores are created in GHPC to accelerate ion transport for higher rate performance almost without the sacrifice of high specific surface area. (b) To further enhance the power density, herein, the graphene has successfully been incorporated into 3D HPC frameworks. This further helps to build good electrical connection with the high-conducting graphene (Fig. 1), remarkably improving the electrical conductivity, verified by electrochemical impedance spectroscopy (EIS) results (Fig. 4e). We find that the GHPC demonstrates a lower equivalent resistance (4.2 Ω) than the HPC (5.2 Ω) and PG (5.6 Ω). We also analyze the dependence of the phase angle on the frequency for the GHPC (Fig. 4f). At low frequencies, the phase angle is about −85°, which is close to ideal capacitive behavior. The characteristic frequency for a phase angle of −45° is 1.1 Hz, which is much higher than that of an activated carbon (0.05 Hz)^26^. It is appealing that the corresponding time constant is as low as 900 ms compared with activated carbons (10 s)^27^,^28^. Such rapid frequency response for our GHPC also leads to the improved rate performance due to the introduction of graphene. (c) The rich heteroatoms (N, O) endow the nanohybrids with high specific capacitance by introducing additional pseudo-capacitance as well as wettability between electrode materials and electrolyte.

**Figure 5:**
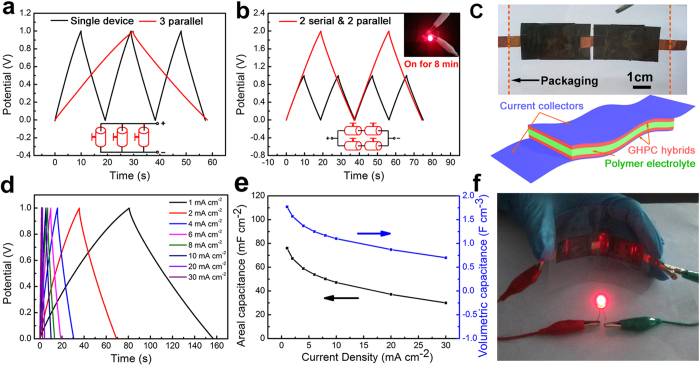
Galvanostatic charge/discharge curves of (a) three supercapacitors assembled in parallel and (b) four supercapacitors connected by combining two in series and two in parallel, the inset showing that the corresponding supercapacitors can be used to power a LED for 8 min; (c) optical photograph of two flexible, all-solid-state GHPC-based supercapacitors connected in series and a schematic diagram of the all-solid-state supercapacitor, (d) the charge and discharge curves and (e) areal and volumetric capacitances as a function of current density for one flexible all-solid-state GHPC-based supercapacitor; (f) a red LED lightened by two flexible supercapacitors connected in series under bending state.

In order to meet the energy and power requirements of portable equipment, supercapacitors need to be packaged together either in series, in parallel, or in combination of the two considering that energy density of a single supercapacitor is too low for better practical applications. The performance of a set of GHPC-based supercapacitors (~1.44 mg of GHPC active materials for one unit) was evaluated by assembling three devices in parallel configuration. A three-fold increase in the output current was obtained (Figure S11a), and hence the discharge time was three times that of a single device under the same current density (Fig. 5a). Furthermore, the four supercapacitors were also connected by combining two in series and two in parallel, as shown in Fig. 5b and Figure S11b. It can be seen that both output potential and discharge time increased by a factor of 2 at the same charge and discharge current, indicating that superior capacitive properties with minimal internal resistance. As a demonstration, such assembled supercapacitors possess the ability to light a red light-emitting diode (LED) with a minimum operating turn-on potential of 1.6 V for as long as 8 min (inset of Fig. 5b, Movie S1).

With the purpose to demonstrate the applications of such fascinating GHPC nanohybrids in flexible devices, we replaced the traditional liquid electrolyte with poly(vinyl alcohol) (PVA)-H_2_SO_4_ polymer gel electrolyte (also acting as the separator) and then assembled a flexible all-solid-state supercapacitor based on our GHPC (detailed preparation process in Supporting Information). Figure 5c shows the two devices in series configuration. The electrochemical performance of each device has been further evaluated, which exhibits typical capacitive behavior with nearly rectangular CV curves at 20–400 mV s^−1^ (Figure S12a), similar to the results in Fig. 4a. Figure 5d gives the corresponding charge and discharge curves at current densities of 1–30 mA cm^−2^, showing typically perfect triangular morphologies. The areal and volumetric capacitances are calculated according to the discharge curves, as shown in Fig. 5e. The areal and volumetric capacitances of the all-solid-state supercapacitors are 76.1 mF cm^−2^ and 1.8 F cm^−3^ at 1 mA cm^−2^, and 30.0 mF cm^−2^ and 0.7 F cm^−3^ at 30 mA cm^−2^. The areal capacitance is much higher than the laser-scribed graphene-based electrochemical capacitors (3.7 mF cm^−2^ at 1 A g^−1^)^27^. In addition, all-solid-state supercapacitor maintains 83% of its initial capacitance after 5000 cycles (Figure S12d). More significantly, the two tandem all-solid-state supercapacitors can be fully charged to 2 V within 10 s and still lighten a LED even in bending state, as shown in Fig. 5f. In a word, these interesting results indicate that the as-synthesized GHPC is one of the most efficient electrode materials for supercapacitors.

In summary, we demonstrate a new protocol for the controlled synthesis of GHPC nanohybrids by utilizing the inherent meso/macroporous architecture and huge absorption ability to water of auricularia biomass. The as-prepared GHPC exhibits much high specific surface area (up to 1723 m^2^ g^−1^), rich mesoporosity (~75%) and much larger pore volume (up to 1.85 cm^3^ g^−1^) than HPC (1.48 cm^3^ g^−1^) and PG (0.66 cm^3^ g^−1^). As a result, the GHPC demonstrates a high specific capacitance of 256 F g^−1^ at 1 A g^−1^ with excellent rate capability (120 F g^−1^ at 50 A g^−1^) and long cycle life (92% capacity retention after 10000 cycles) for symmetric supercapacitors in 1 M H_2_SO_4_. The excellent electrochemical performance is at least comparable to the best reports in the literature for two-electrode configuration in aqueous solution. Furthermore, our GHPC can be used to construct flexible and all-solid-state supercapacitor in parallel or series even combining parallel with series, which can be fully recharged within 10 s and able to light an LED even under bended state based on the rational assembly. Our work demonstrates the important role of a balanced mesoporosity to microporosity on enhancing the performance of supercapacitors, and may open a new avenue for the development of advanced carbon nanomaterials with special pores for the future supercapacitors.

## Methods

### Preparation of the GHPC nanohybrids

The GO was synthesized using a modified Hummer’s method and aqueous dispersion of GO with a concentration of 5.0 mg mL^−1^ was made by prolonged stir and ultrasonication. The GHPC was prepared according to the following protocols. Typically, a 20 mL of GO solution and 3.0 g of auricularia were placed in a 50 mL teflon-lined autoclave and maintained for several hours until the volume of the auricularia expanded completely. The autoclave was sealed and heated at 180 °C for 12 h and then cooled to room temperature naturally. The obtained hydrothermal products were collected by filtration, washed with distilled water, and dried. Subsequently, the hydrothermal products (0.3 g) were dispersed and stirred in KOH solution (30 mL, 0.03 g mL^−1^) for 2 h. Then the suspension liquid was transferred into dish at 80 °C oven overnight till a black powder was formed. The black powder was then heated under Ar atmosphere with a heating rate of 3 °C min^−1^ to 850 °C for 2 h. After that, the activated samples were thoroughly washed with 10 wt % HCl and distilled water. Finally, the product was dried in an oven at 60 °C to obtain GHPC. As a comparison, the hierarchical porous carbon (HPC) has been synthesized using the same strategy simply without the addition of GO. And the KOH-activated porous graphene (PG) was also prepared by activating hydrothermally reduced GO.

### Characterizations

The morphologies were characterized by field emission SEM (S-4800) and TEM (JEOL 2100). The porous properties were analyzed using nitrogen adsorption and desorption isotherms that were obtained using the surface area and a porosimetry analyzer (ASAP 2010) at 77 K. The specific surface areas were calculated according to the BET theory. PSD was calculated via BJH method. The structure was checked by XRD (Rigaku D/max 2550VB/PC), Raman spectra (Renishaw inVia Reflex). XPS (PHI 5700 ESCA) was performed using monochromated Al Kα radiation. Elemental analysis was determined by Elementar Vario III (Germany).

### Electrochemical Measurements

The active materials (GHPC, HPC and PG) were mixed with acetylene black and PVDF (80:10:10, w/w/w) in NMP solution to form sticky slurry. For a two-electrode cell configuration, the slurry was coated on the graphite paper disk with an area of about 1.13 cm^2^ and a mass loading of active material of ~0.7 mg cm^−2^ as working electrode. Then, two identical electrodes were sandwiched with a polypropylene (PP) separator and 1 M aqueous H_2_SO_4_ as electrolyte using a teflon swagelok type two-electrode configuration with two stainless-steel sheets. In a three-electrode cell, the working electrode was prepared by coating the slurry onto one end of the graphite paper (~1 cm^2^) after dried at 120 °C in vacuum. The amount of active materials on each current collector was ~0.8 mg. The three-electrode system was tested in 1 M H_2_SO_4_ with a Pt foil as the counter electrode and a saturated calomel electrode (SCE) as the reference electrode. All the electrochemical measurements were carried out on an Autolab PGSTAT30 electrochemical workstation. The EIS test was conducted in the same electrolyte with a frequency loop from 100 kHz to 10 mHz using a perturbation amplitude of 5 mV.

## Additional Information

**How to cite this article**: Zhu, Z. *et al.* Dual Tuning of Biomass-Derived Hierarchical Carbon Nanostructures for Supercapacitors: the Role of Balanced Meso/Microporosity and Graphene. *Sci. Rep.* **5**, 15936; doi: 10.1038/srep15936 (2015).

## Supplementary Material

Supporting Information

Supplementary Movie S1
