# Supplementary material for: Dual Tuning of Biomass-Derived Hierarchical Carbon Nanostructures for Supercapacitors: the Role of Balanced Meso/Microporosity and Graphene
Source: Sci Rep. 2015 Oct 30;5:15936. doi: 10.1038/srep15936 (PMC4626760; doi:10.1038/srep15936)
Supplement: Supporting Information [file srep15936-s1.doc]

[**Supporting information**](http://www.nature.com/nmat/journal/v8/n7/suppinfo/nmat2469_S1.html)

Dual Tuning of Biomass-Derived Hierarchical Carbon Nanostructures for Supercapacitors: the Role of Balanced Meso/Microporosity and Graphene

Zhengju Zhu^1^, Hao Jiang^1^*, Shaojun Guo^2^, Qilin Cheng^1^, Yanjie Hu^1^, Chunzhong Li^1^*

^1^ Key Laboratory for Ultrafine Materials of Ministry of Education, School of Materials Science and Engineering, East China University of Science and Technology, Shanghai 200237, China. * E-mail: [jianghao@ecust.edu.cn](mailto:jianghao@ecust.edu.cn) (Prof. H. Jiang), [czli@ecust.edu.cn](mailto:czli@ecust.edu.cn) (Prof. C. Z. Li); Tel: +86-21-64250949, Fax: +86-21-64250624

^2^ Physical Chemistry and Applied Spectroscopy, Los Alamos National Laboratory, Los Alamos, New Mexico 87545, United States.

**Part I: Experimental Section**

**Preparation of the polymer gel electrolyte (PVA-H_2_SO_4_).** The gel electrolyte was prepared according to the method described in reference.^28^

**Fabrication of flexible all-solid-state GHPC-based supercapacitors.** Sticky slurry containing GHPC was prepared using the same method described in the main text. The graphite paper was cut into a rectangle with desirable size. Then the slurry was coated on the graphite paper with square area and a mass loading of active material of ~ 0.8 mg cm^-2^ after drying in vacuum. A narrow strip without slurry loading on edge of graphite paper was glued to copper foil to ensure good electrical contact between supercapacitors and the electrochemical workstation. The gel electrolyte was drop-cast slowly on the electrode area, and left under ambient conditions for 5 hours for complete infiltration of electrolyte and evaporation of any excess water. The supercapacitor was assembled by two electrodes face-to-face and left overnight until the electrolyte solidified. Finally, the as-fabricated supercapacitor was sealed by flexible thermoplastics using heat-seal apparatus.

**Part II:** **Calculations**

The specific gravimetric capacitance for a single electrode in 1 M H_2_SO_4_ aqueous solution was calculated from the discharge curves by using the formula of $C=2It/m\Delta V$ for the two-electrode cells, while $C=It/m\Delta V$ for the three-electrode system, where $I$ (A) is the charge/discharge current and $m$ (g) is the mass of active materials on single electrode, $t$ (s) is the discharge time and $\Delta V$ (V) is the voltage change during the discharge process. Besides, the volumetric specific capacitance based on the entire electrode volume (active materials, acetylene black and PVDF) was calculated using the formula for three-electrode system:

$C_{v}=C_{electrode}m/V_{eletrode}$ (1)

Where $C_{v}$ (F cm^-3^) is the volumetric specific capacitance for one electrode, $C_{electrode}$ is the gravimetric specific capacitance, $m$ (g) is the mass of active materials on single electrode and $V_{electrode}$ is the entire electrode volume. The total thickness of the electrode is ~ 50 µm.

The capacitance of flexible all-solid-state supercapacitors was calculated from the galvanostatic discharge curves using the formula:

$C=It/\Delta V$ (2)

Where $C$ (F) is the capacitance, $I$ (A) is the charge/discharge current, $t$ (s) is the discharge time and $\Delta V$ (V) is the voltage change. The gravimetric, areal and volumetric specific capacitances of supercapacitor were calculated according to the following formulae:

*Gravimetric specific capacitance=*$C/M$ (3)

*Areal specific capacitance=*$C/A$ (4)

*Volumetric specific capacitance=*$C/V$ (5)

where $M$ (g), $A$ (cm^2^) and $V$ (cm^3^) refer to total mass of electroactive materials, the area and the volume of an all-solid-state supercapacitor, respectively. The volumetric capacitance (F cm^-3^) was calculated taking into account the whole volume of the active materials, the current collectors and the solidified electrolyte, and it did not include the packaging. This total volume avoids the pitfall of studies that report high volumetric energy and power densities by using thin layer of active material on comparatively thick current collectors and electrolyte. The total thickness of the supercapacitor (without the packaging) is ~ 430 µm.

**Part III: Supporting Figures**


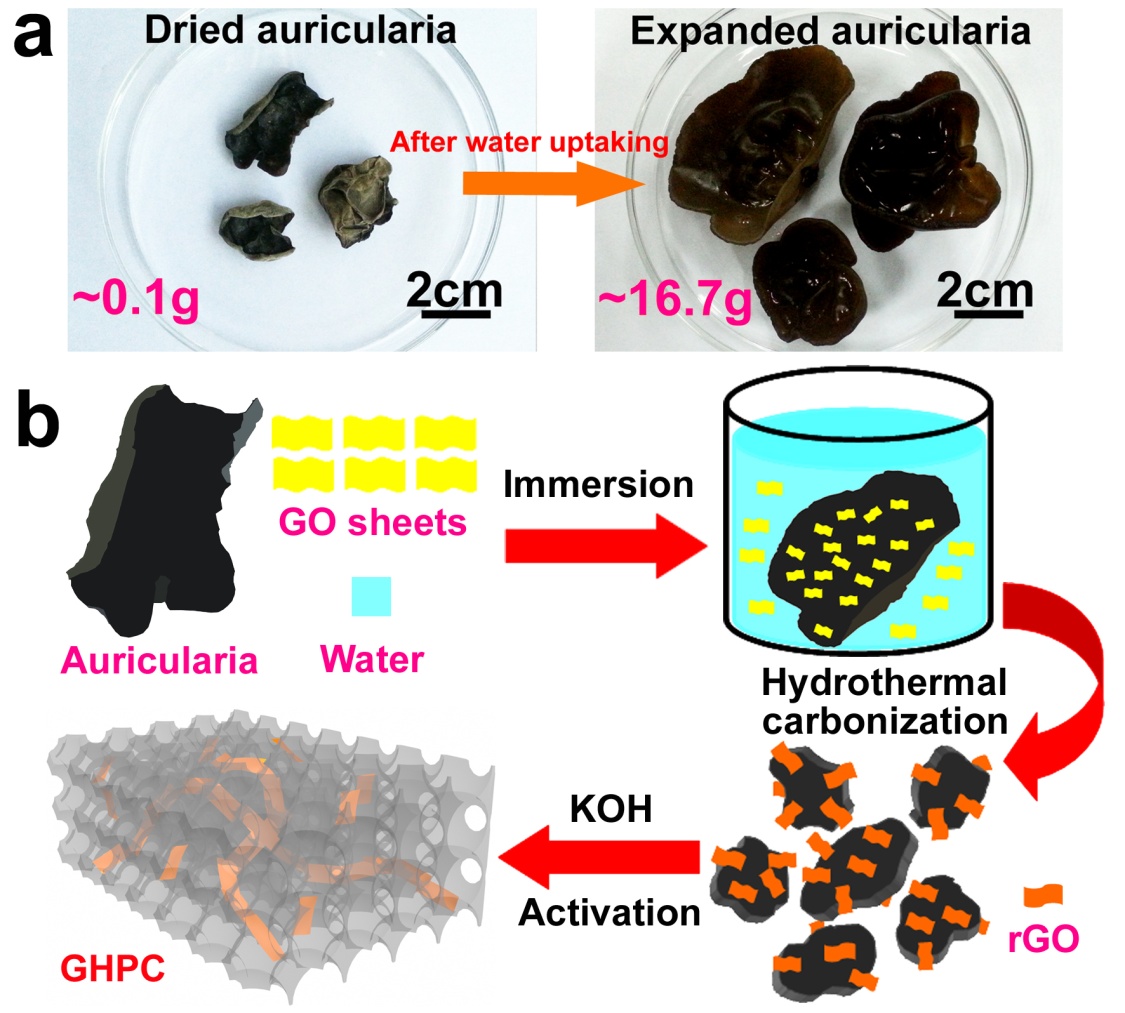


**Figure S1 |** The Photograph and schematic illustration. (a) The photographs of the auricularia before and after immersion in water. (b) Schematic illustration of the methodology strategy.


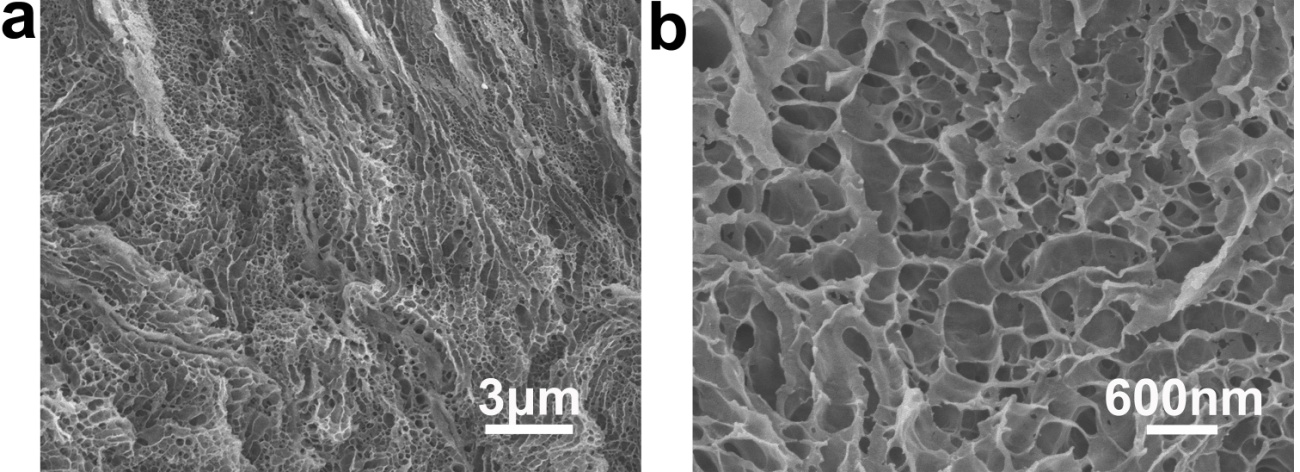


**Figure S2 |** (a) Low- and (b) high-magnification SEM images of the cross-section of the dried auricularia.


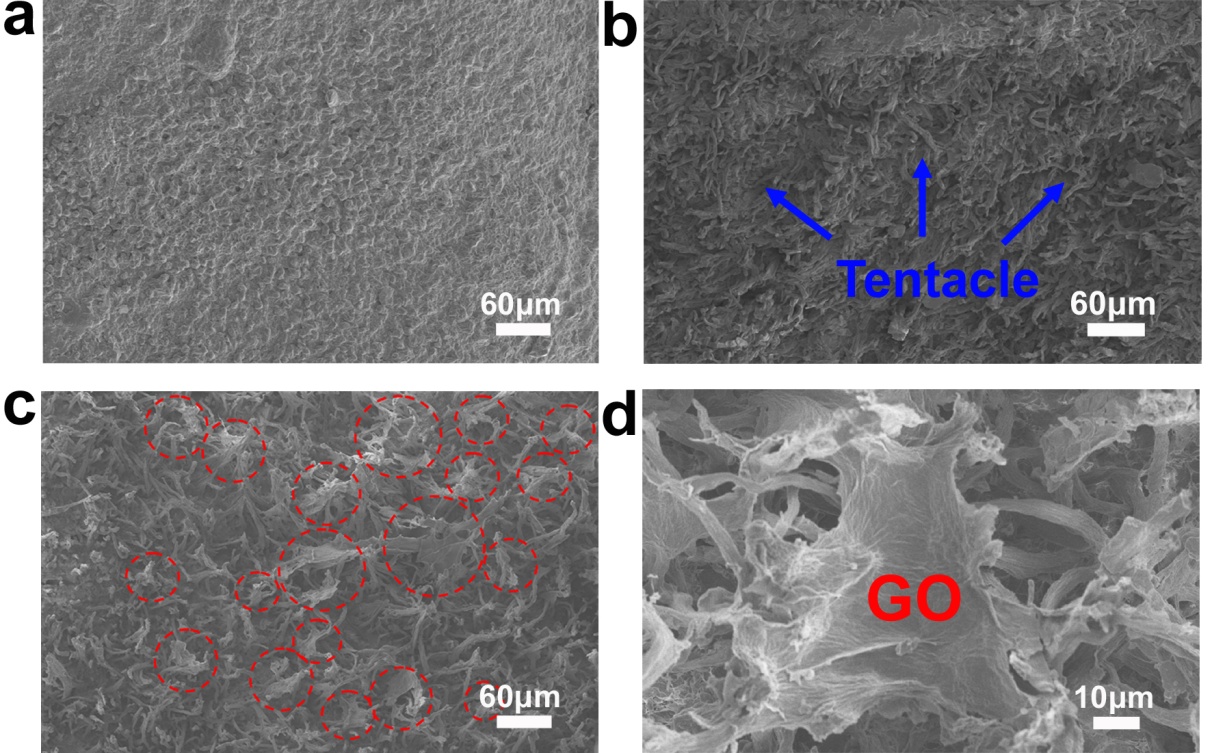


**Figure S3 |** SEM images of the auricularia before and after immersion in GO solution. (a, b) The undulated surface of the auricularia with wrinkles (a) and tentacles (b) before immersion. (c, d) The surface with tentacles after immersion, red dotted line areas in (c) display GO anchored on the tentacles.


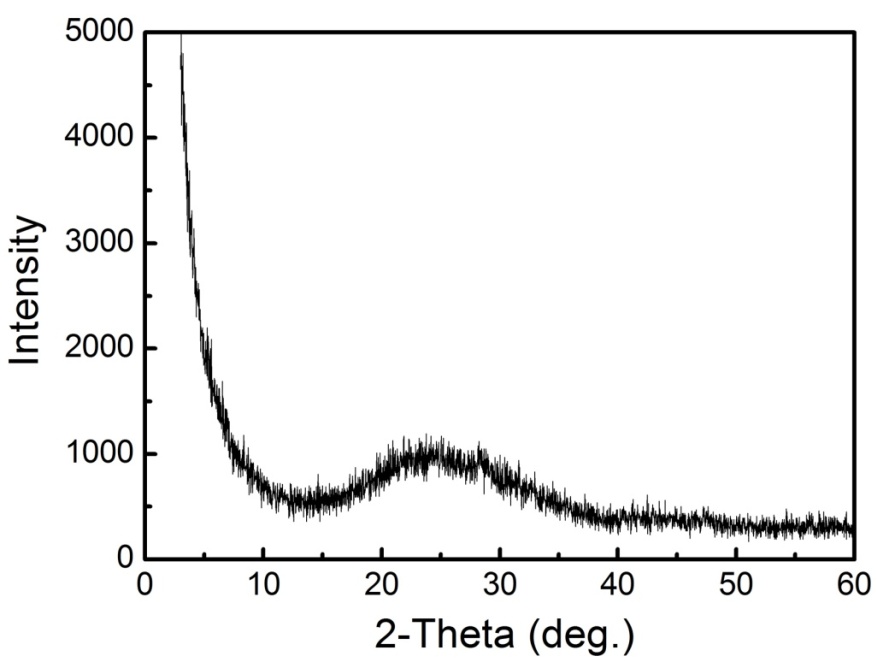


**Figure S4 |** Low and wide angle XRD pattern of the GHPC. A large intensity increase in the low-angle scatter suggests the presence of micropores and mesopores.


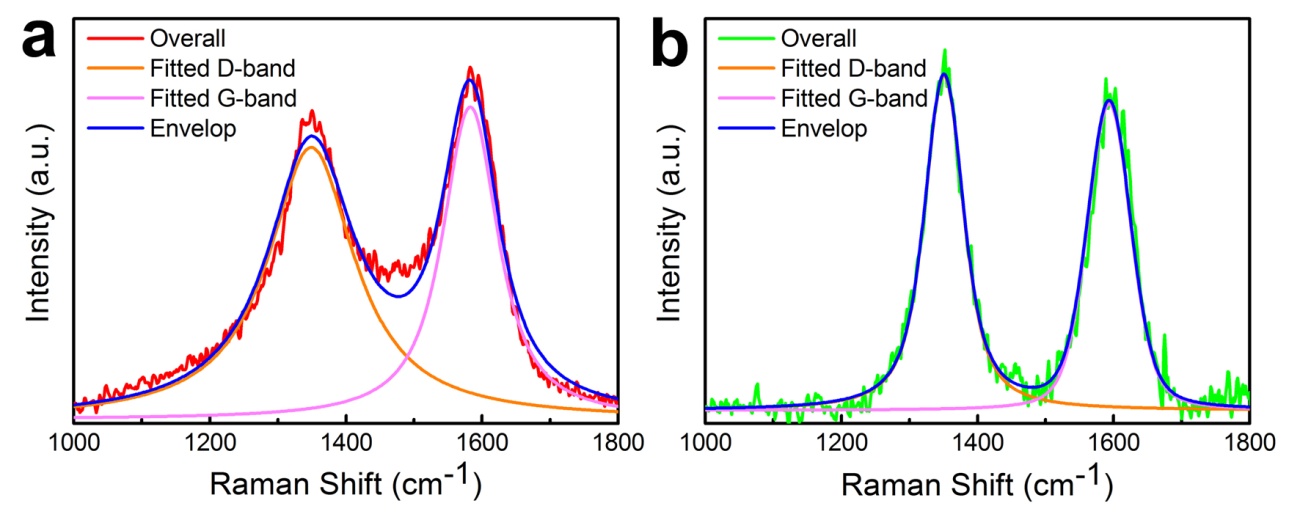


**Figure S5 |** The fitted Raman curves. (a) GHPC, (b) PG. The fitted Raman curves were generated by simulating the original curves using voigt function.


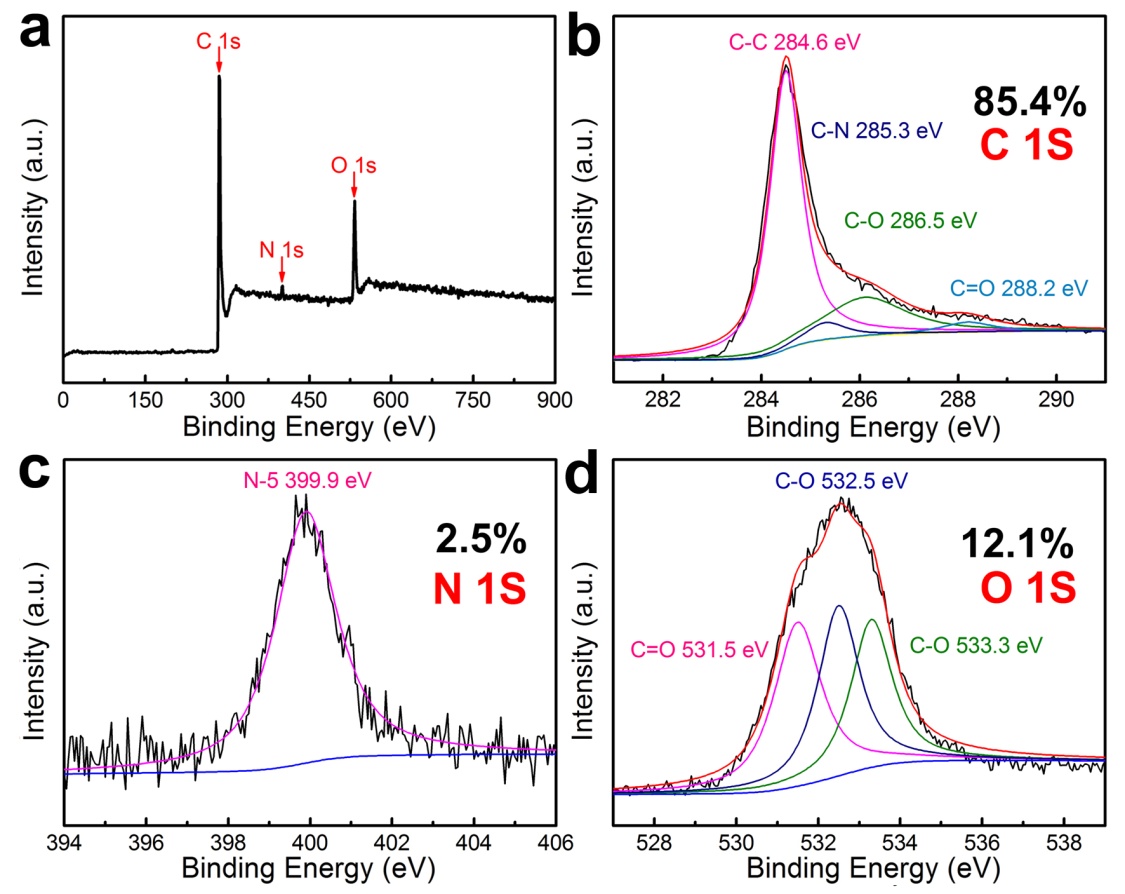


**Figure S6 |** XPS spectra of the GHPC. (a) Survey, (b) C 1s, (c) N 1s, (d) O 1s.


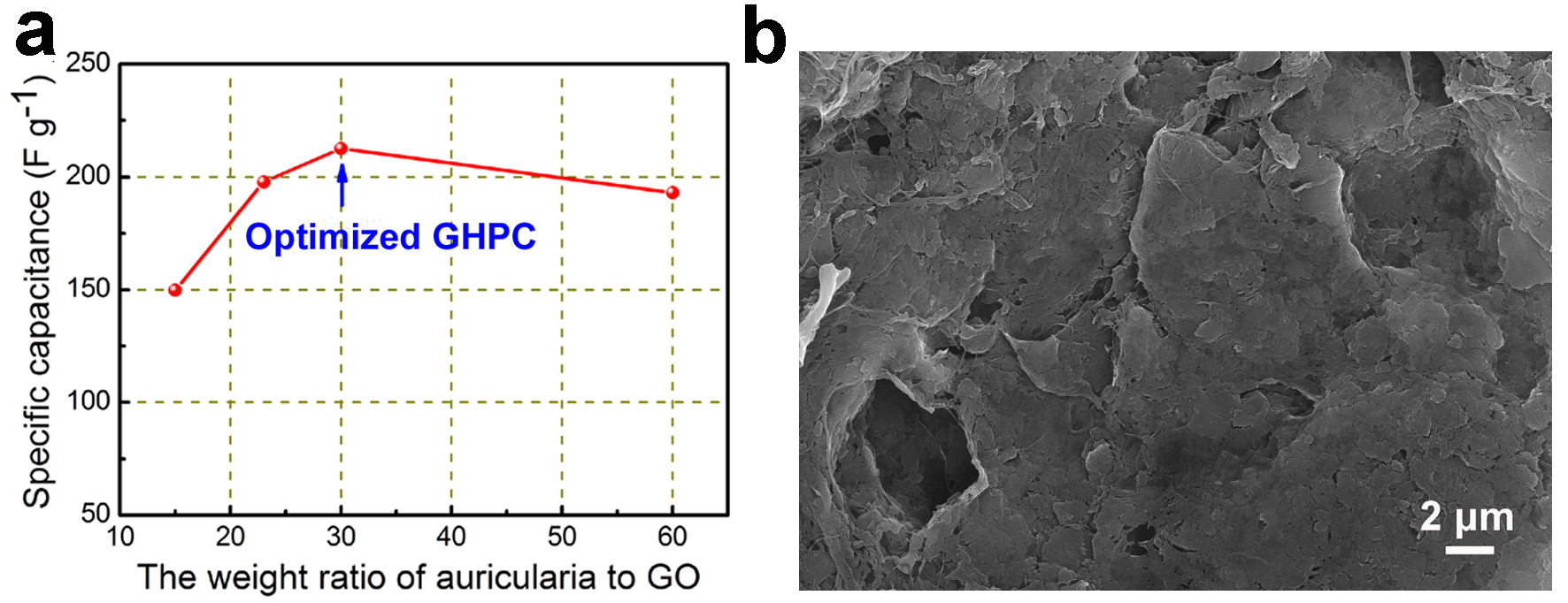


**Figure S7 |** (a) The specific capacitance as a function of the weight ratio of auricularia to GO at 20 A g^-1^ in a three-electrode system, (b) SEM image of GHPC-15 (the weight ratio of auricularia to GO is 15).

The highest specific capacitance of GHPC is achieved when the weight ratio of auricularia to GO is 30 (GHPC-30). At a low graphene content, *e.g.* GHPC-60 (the weight ratio of auricularia to GO is 60), the specific capacitance is 193 F g^-1^ at 20 A g^-1^ in the three-electrode system, slightly higher than the corresponding HPC. Increasing the graphene content, the specific capacitance is also gradually increased. But, when more graphene nanosheets have been added, *e.g.* GHPC-15 and GHPC-23, some of them are hard to be incorporated into the HPC frameworks and stack on the outer surface, which may inhibit the full contact of interior active materials with the electrolyte, as shown in SEM images (Figure S7b). Therefore, it is necessary to optimize the composition of GHPC hybrids.


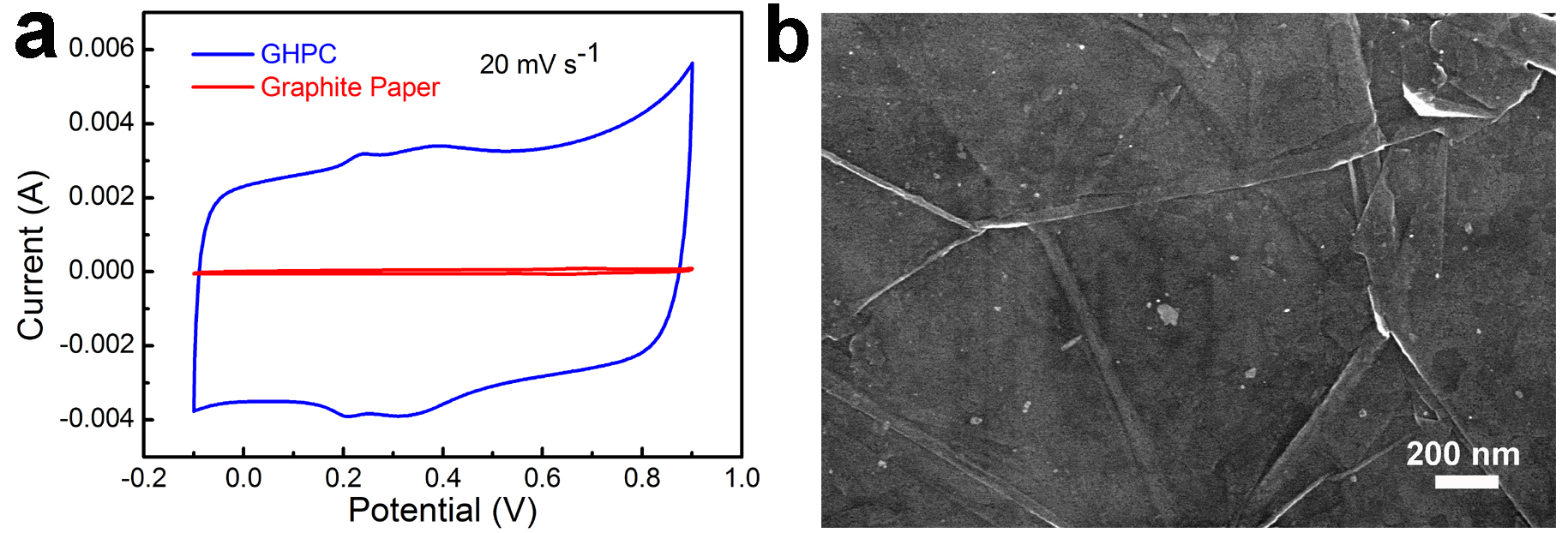


**Figure S8 |** (a) CV curves of the as-obtained GHPC and graphite paper at 20 mV s^-1^ in H_2_SO_4_ electrolyte in the three-electrode system, (b) SEM image of graphite paper.


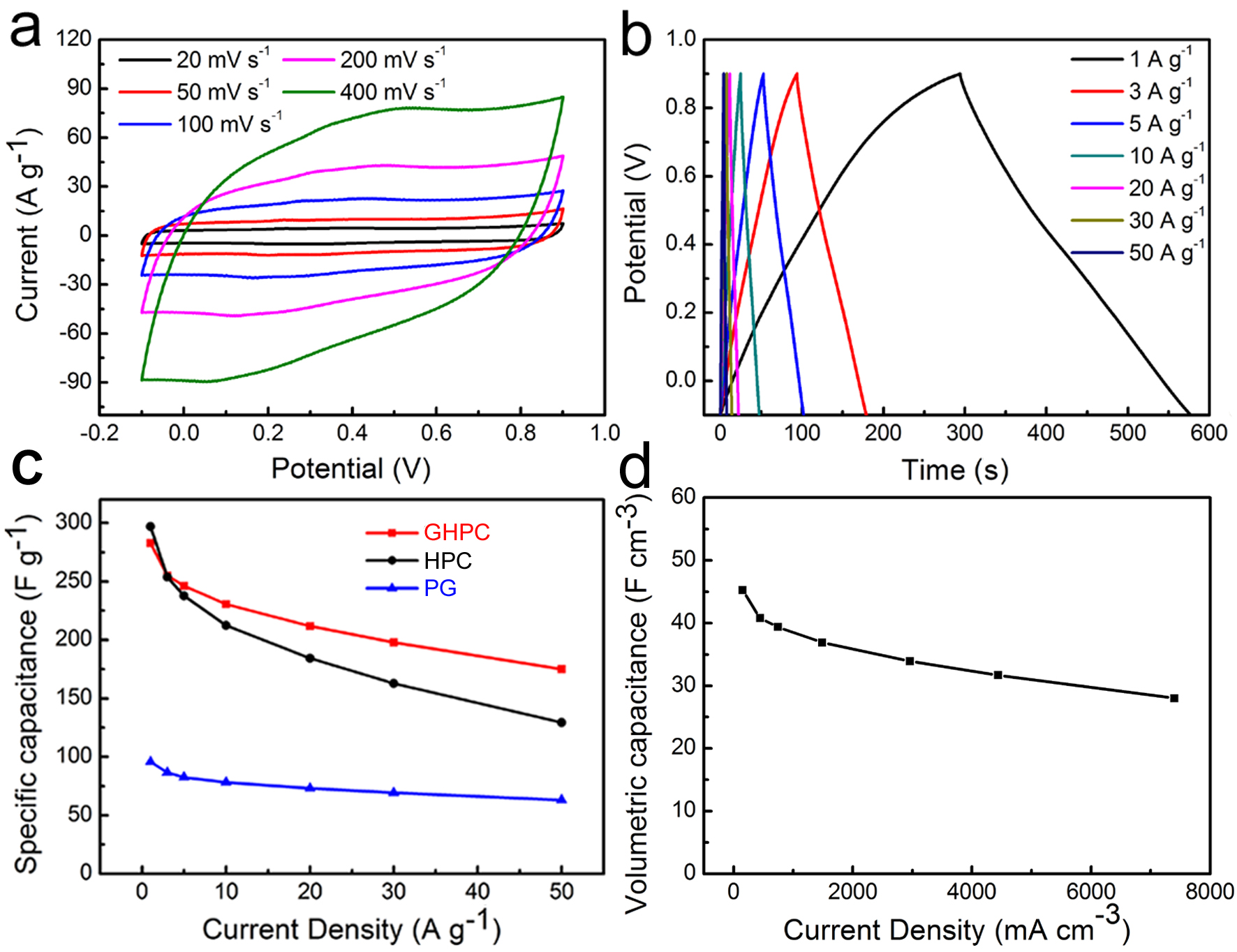


**Figure S9 |** Electrochemical performance of the GHPC in a three-electrode system using 1 M H_2_SO_4_ aqueous solution. (a) CV curves of the GHPC at various scan rates, (b) Galvanostatic charge/discharge curves of the GHPC under different current densities, (c) The specific capacitance as a function of the current densities, (d) The volumetric specific capacitance as a function of the current density.

All CV curves display quasi-rectangular shapes with some humps, suggesting a contribution from pseudo-capacitance from not only the O functionalities but also the N functionalities (Figure S6). These redox peaks are no observed in the two-electrode system, which is consistent with previous reports.^19,23^ The specific capacitance of the GHPC calculated by the galvanostatic charge/discharge is 282.9 F g^-1^ at a current density of 1 A g^-1^ and 175.0 F g^-1^ at a current density of 50 A g^-1^ (Figure S9b), revealing a better rate capability than other two controlled samples (Figure S9c), which is mainly due to the interconnected ion channels existing in the material, and also the good intrinsic electronic properties derived from incorporated graphene. Moreover, the volumetric specific capacitances based on all electrode volume are calculated as shown in Figure S9d. The volumetric specific capacitances of the GHPC electrode are 45.3 F cm^-3^ at 148 mA cm^-3^ and 28.0 F cm^-3^ at 7400 mA cm^-3^.


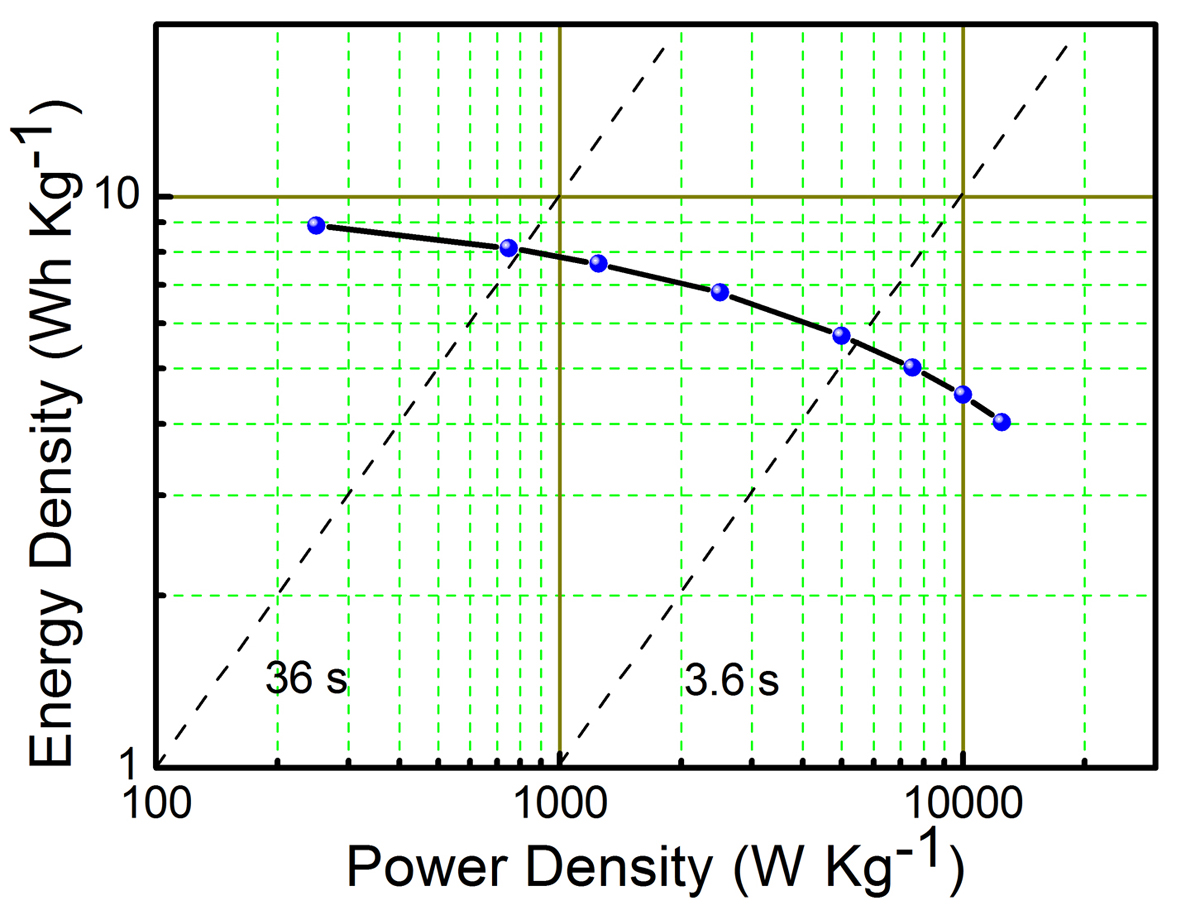


**Figure S10 |** Ragone plot (energy density vs. power density) of GHPC-based supercapacitor in H_2_SO_4_ electrolyte. The energy density and power density were estimated by using the formula: $E=C_{electrode}V^{2}/8$ and $P=E/t$, where $C_{electrode}$ is specific capacitance of one electrode.


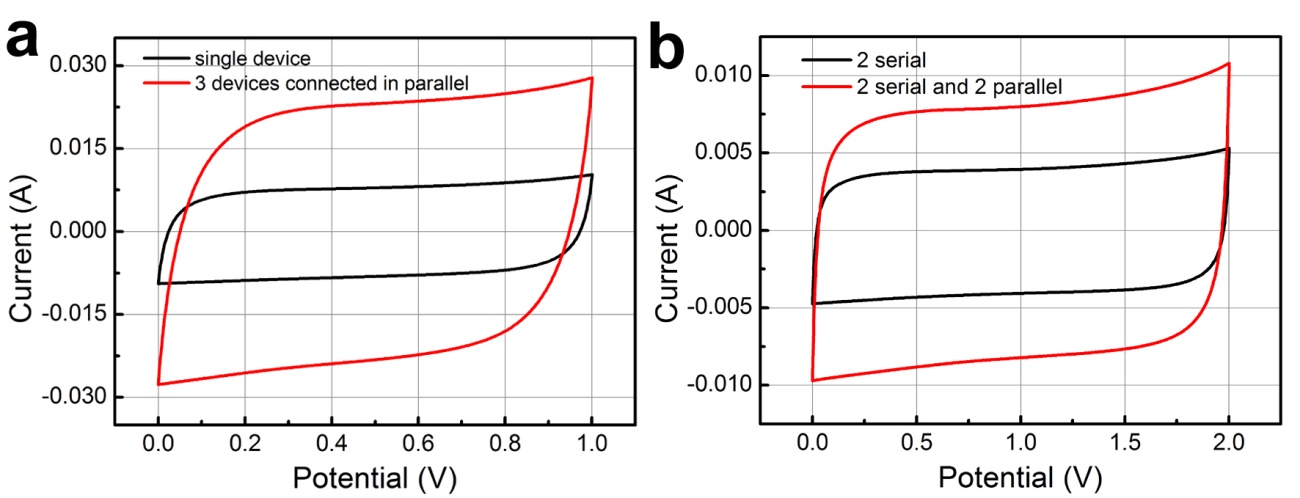


**Figure S11 |** (a, b) CV curves of the optimized GHPC at 200 mV s^-1^ for three supercapacitors connected in parallel (a) and four supercapacitors connected in a combination of series and parallel (b).


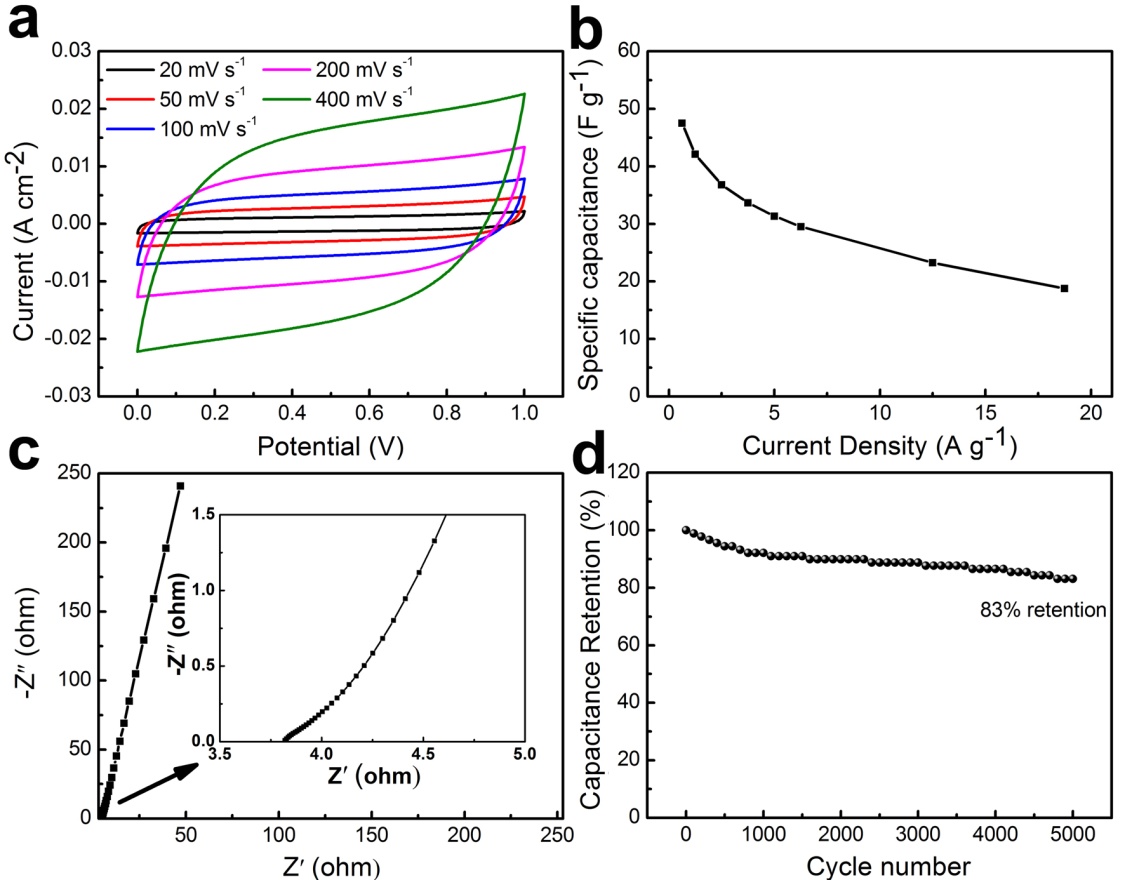


**Figure S12** **|** Electrochemical performance of a flexible, all-solid-state GHPC-based supercapacitor. **(**a) CV curves at various scan rates, (b) the specific capacitance as a function of the current densities (based on total mass of electroactive materials in a flexible supercapacitor), (c) the Nyquist plot and (d) the cycling stability test at 6 mA cm^-2^ for the all-solid-state GHPC-based supercapacitor.


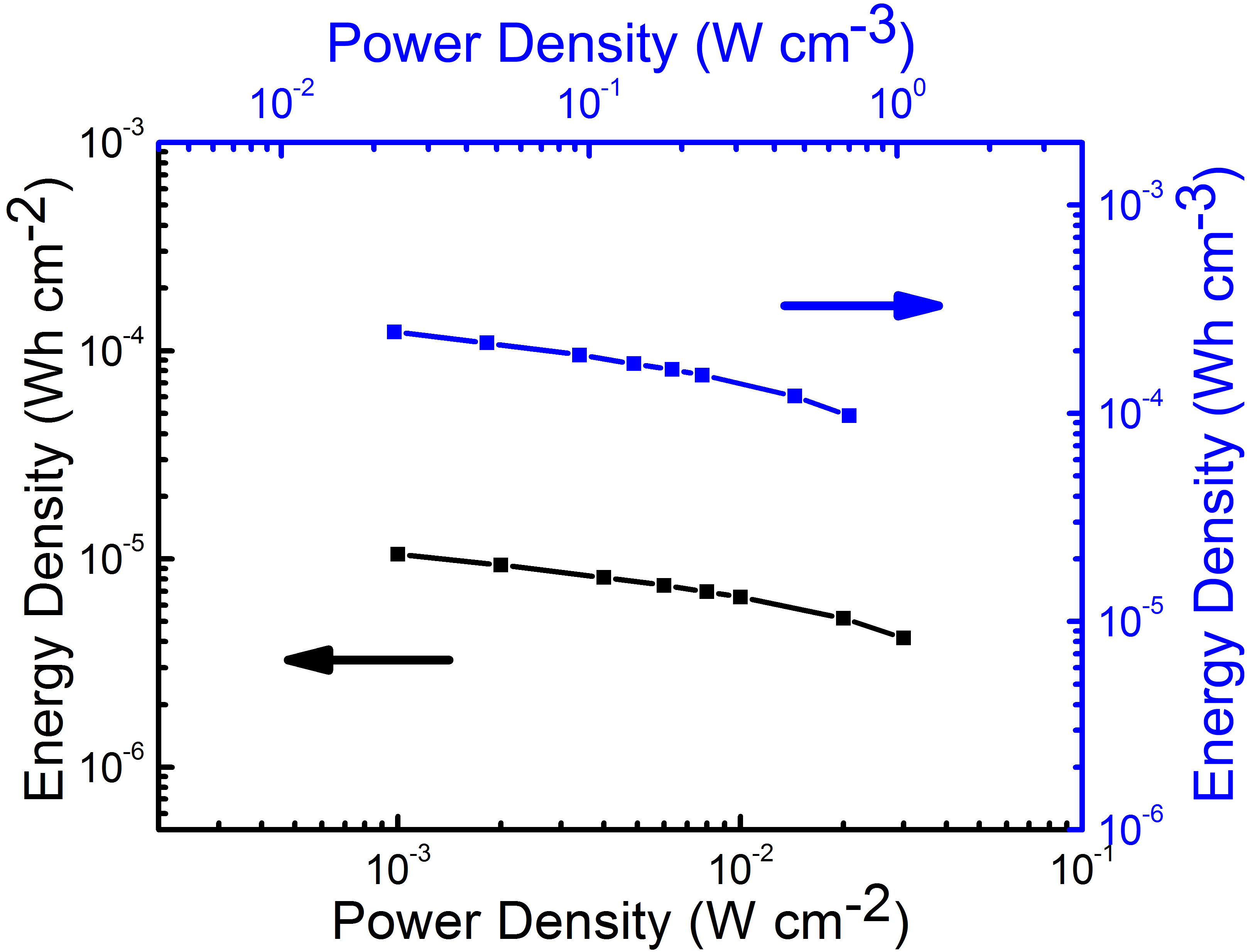


**Figure S13 |** Ragone plot (energy density vs. power density) of the flexible, all-solid-state GHPC-based supercapacitor. The energy density and power density were estimated by using the formula: $E=C_{\sup ercapacitor}V^{2}/2$ and $P=E/t$, where $C_{\sup ercapacitor}$ is areal (volumetric) specific capacitance of one supercapacitor.

**Part IV: Movie**

**Movie S1** **|** Practical application of the GHPC supercapacitors using 1 M H_2_SO_4_ aqueous solution. Four supercapacitors were connected in a combination of series and parallel as shown in Figure 5b in this movie. After charging this system at a constant potential of 2.0 V, it is used to light up a red LED (with a minimum operating potential of 1.6 V) for about 8 minutes.

**Part V: Supporting Table**

**Table S1 |** Comparison of the electrochemical performance of carbon materials in aqueous electrolyte under two-electrode system reported in literature.

| Samples | Specific surface area (m^2^ g^-1^) | Electrolyte | Current density (A g^-1^) | Capacitance (F g^-1^) | Ref. |
| --- | --- | --- | --- | --- | --- |
| Silk-derived microporous carbon nanoplates | 2557 | 1 M H_2_SO_4_ | 0.1 | 264 | 24 |
|  |  |  | 52.5 | 120 |  |
| N, P-co-doped carbon nanofibers | 290 | 2 M H_2_SO_4_ | 1 | 205 | 25 |
|  |  |  | 50 | 125 |  |
| 3D hierarchical porous carbon | 2870 | 6 M KOH | 2 | 236 | 9 |
|  |  |  | 30 | 182 |  |
| Dead leaves-derived microporous carbon | 1230 | 1 M H_2_SO_4_ | 1 | 302 | S1 |
|  |  |  | - | - |  |
| Carbonized eggshell membrane | 221 | 1 M H_2_SO_4_ | 2 | 205 | 23 |
|  |  |  | - | - |  |
| Seaweed-derived porous carbon | 746 | 1 M H_2_SO_4_ | 0.2 | 264 | S2 |
|  |  |  | - | - |  |
| Sunflower seed shell-derived carbon | 1162 | 30 wt% KOH | 0.25 | 244 | S3 |
|  |  |  | 10 | 171 |  |
| Reduced GO foams | - | 1 M H_2_SO_4_ | 0.5 | 117 | S4 |
|  |  |  | - | - |  |
| Folded graphene paper | - | 1 M H_2_SO_4_ | 1 | 172 | S5 |
|  |  |  | 50 | 135 |  |
| N-doped graphene | - | 6 M KOH | 1 | 282 | S6 |
|  |  |  | 33 | 165 |  |
| GHPC | 1723 | 1 M H_2_SO_4_ | 1 | 256 | This work |
|  |  |  | 50 | 120 |  |

**Table S2 |** Comparison of the energy density of carbon materials under two-electrode system reported in literatures.

| Samples | Electrolyte | C_g_  (F g^-1^) | E_g_  (Wh kg^-1^) | Ref. |
| --- | --- | --- | --- | --- |
|
| Seaweeds-derived carbon | 1 M H_2_SO_4_ | 198 | 7.4 | S7 |
| High density porous graphene | 6 M KOH | 260 | 8.3 | S8 |
| 3D microporous carbon | 1 M H_2_SO_4_ | 255 | 8.8 | S9 |
| Hexaporous carbon | 1 M H_2_SO_4_ | 154 | 5.3 | S10 |
| N-doped carbon fiber | 6 M KOH | 202 | 7.1 | S11 |
| Macroporous graphene | 1 M Na_2_SO_4_ | 202 | 5 | S12 |
| Graphene/carbon black | 6 M KOH | 138 | 5 | S13 |
| GHPC | 1 M H_2_SO_4_ | 256 | 8.9 | This work |

**References**

S1. Biswal, M., Banerjee, A., Deoab, M. & Ogale, S. From dead leaves to high energy density supercapacitors. *Energy Environ. Sci.* **6**, 1249-1259 (2013).

S2. Raymundo-Pinero, E., Cadek, M. & Beguin, F. Tuning carbon materials for supercapacitors by direct pyrolysis of seaweeds. *Adv. Funct. Mater.* **19**, 1032-1039 (2009).

S3. Li, X. *et al*. Preparation of capacitor's electrode from sunflower seed shell. *Bioresour. Technol.* **102**, 1118-1123 (2011).

S4. Niu, Z. Q., Chen, J., Hng, H. H., Ma, J. & Chen, X. D. A leavening strategy to prepare reduced graphene oxide foams. *Adv. Mater.* **24**, 4144-4150 (2012).

S5. Liu, F., Song, S. Y., Xue, D. F. & Zhang, H. J. Folded structured graphene paper for high performance electrode materials. *Adv. Mater.* **24**, 1089-1094 (2012).

S6. Jeong, H. M. *et al*. Nitrogen-doped graphene for high-performance ultracapacitors and the importance of nitrogen-doped sites at basal planes. *Nano. Lett.* **11**, 2472-2477 (2011).

S7. Raymundo-Pinero, E., Leroux, F. & Beguin, F. A high-performance carbon for supercapacitors obtained by carbonization of a seaweed biopolymer. *Adv. Mater.* **18**, 1877-1882 (2006).

S8. Tao, Y. *et al*. Towards ultrahigh volumetric capacitance: graphene derived highly dense but porous carbons for supercapacitors. *Sci. Rep.* **3**, 2975-2982 (2013).

S9. Puthusseri, D., Aravindan, V., Srinivasan, M. & Ogale, S. 3D micro-porous conducting carbon beehive by single step polymer carbonization for high performance supercapacitors: the magic of *in situ* porogen formation. [*Energy Environ. Sci.*](http://dx.doi.org/10.1039/1754-5706/2008) **7**, 728-735 (2014).

S10. Yadav, P. *et al.* A 3D hexaporous carbon assembled from single-layer graphene as high performance supercapacitor. *Chemsusche*m **5**, 2159-2164 (2012).

S11. Chen, L. F. *et al*. Synthesis of nitrogen-doped porous carbon nanofibers as an efficient electrode material for supercapacitors. *ACS Nano* **6**, 7092-7102 (2012).

S12. Choi, B. G., Yang, M., Hong, W. H., Choi, J. W. & Huh, Y. S. 3D macroporous graphene frameworks for supercapacitors with high energy and power densities. *ACS Nano* **6**, 4020-4028 (2012).

S13. Wang, G. K. *et* *al*. Flexible pillared graphene-paper electrodes for high-performance electrochemical supercapacitors. *Small* **8**, 452-459 (2012).
